# Identification of CpG islands in DNA sequences using statistically optimal null filters

**DOI:** 10.1186/1687-4153-2012-12

**Published:** 2012-08-29

**Authors:** Rajasekhar Kakumani, Omair Ahmad, Vijay Devabhaktuni

**Affiliations:** 1 Department of Electrical and Computer Engineering, Concordia University, 1455 de Maisonneuve Blvd. West Montreal, QC H3G1M8, Canada; 2 Department of Electrical Engineering and Computer Science, University of Toledo, MS 308, 2801 W. Bancroft St., Toledo, OH 43606, USA

## Abstract

CpG dinucleotide clusters also referred to as CpG islands (CGIs) are usually located in the promoter regions of genes in a deoxyribonucleic acid (DNA) sequence. CGIs play a crucial role in gene expression and cell differentiation, as such, they are normally used as gene markers. The earlier CGI identification methods used the rich CpG dinucleotide content in CGIs, as a characteristic measure to identify the locations of CGIs. The fact, that the probability of nucleotide G following nucleotide C in a CGI is greater as compared to a non-CGI, is employed by some of the recent methods. These methods use the difference in transition probabilities between subsequent nucleotides to distinguish between a CGI from a non-CGI. These transition probabilities vary with the data being analyzed and several of them have been reported in the literature sometimes leading to contradictory results. In this article, we propose a new and efficient scheme for identification of CGIs using statistically optimal null filters. We formulate a new CGI identification characteristic to reliably and efficiently identify CGIs in a given DNA sequence which is devoid of any ambiguities. Our proposed scheme combines maximum signal-to-noise ratio and least squares optimization criteria to estimate the CGI identification characteristic in the DNA sequence. The proposed scheme is tested on a number of DNA sequences taken from human chromosomes 21 and 22, and proved to be highly reliable as well as efficient in identifying the CGIs.

## Introduction

In the recent years, computational methods for processing and interpreting vast amount of genomic data, generated from genome sequencing, have gained a lot of scientific interest. Genomic sequences such as deoxyribonucleic acid (DNA) consist of biological instructions which are crucial for the development and normal functioning of almost all living organisms [1]. A DNA molecule has a complex double helix structure that involves two strands, consisting of alternating sugar and phosphate groups. Attached to these sugar groups of each DNA strand are one of the four chemical bases, namely, adenine (A), thymine (T), guanine (G), and cytosine (C). A unit comprising of base, sugar, and phosphate is referred to as a nucleotide. Hydrogen bonds between the nucleotides A and T (similarly between nucleotides G and C) from the opposite strands not only stabilize the DNA molecule, but also make the two strands complimentary. Nucleotides in a DNA strand exhibit short, recurring patterns (also called sequence motifs) that are presumed to have a biological function. Identification of these patterns helps in understanding the biological information hidden in a DNA sequence. A human DNA consists of about 3 billion nucleotides and completion of genome sequencing of numerous model organisms has further proliferated genomic databases. To completely decipher, the biological information in a DNA sequence is a daunting task and development of fast, efficient, and cost effective computational techniques for the same is a big challenge.

**Figure 1:**
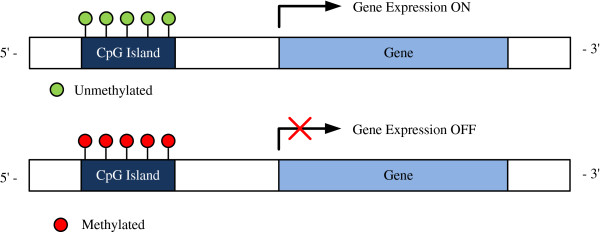
Difference between mythelated and unmythelated CpG Island.

A sequence pattern that plays a crucial role in the analysis of genomes is CpG Island (CGI). A typical CGI consists high-frequency of CpG dinucleoetides, where ‘p’ refers to the phosphodiester bond between the adjacent nucleotides [1,2]. This bond is different from the hydrogen bond that exists between C and G across two strands in a DNA double helix. The length of a CGI varies from a few hundred to a few thousand base pairs (bp), but rarely exceeds 5000 bp. It is known that CpG Islands (CGIs) occur in and around the promoter regions of (50–60)% of human genes, including most housekeeping genes (the genes which are essential for general cell functions) [3]. Gene is a stretch of DNA sequence which has biological information for the synthesis of a protein. The promoter region in a gene regulates its functionality [4-7]. Due to the association of CGIs with promoters, CGIs play an important role in promoter prediction and consequently in the prediction of genes [8,9]. CGIs also contribute significantly in discovering the epigenetic causes of cancer. CGIs located in the promoter regions of certain tumor suppressor genes are normally unmethylated in healthy cells. DNA methylation is a biochemical modification resulting from addition of a methyl group to cytosine nucleotide (C). In cancer cells, CGIs usually undergo a dense hypermethylation leading to gene silencing as shown in Figure 1. Owing to this, they can be used as candidate regions for aberrant DNA methylation, for early detection of cancer [10-14]. For these reasons, identification of CGIs has become indispensable for genome analysis and annotation.

Despite their accuracy, experimental methods employed by biologists for identification of CGIs are extremely time-consuming, simply because of the enormity of genomic data. On the other hand, computational methods can be much more attractive for the identification of possible CGIs. The results obtained from computational methods can be used by biologists to validate and further enhance the accuracy of identified CGI locations. There are several computational methods [15-26] reported in the literature for identification of CGIs in DNA sequences. In one of the first computational attempts [15], a CGI is defined as a DNA segment fulfilling the following three conditions: (i) length of segment is at least 200 bp, (ii) G and C contents are ≥ 50%, and (iii) observed CpG to expected CpG ratio (o/e) is ≥ 0.6. Observed CpG is the number of CpG dinucleoetides in a segment and expected CpG is calculated by multiplying the number of ‘C’s and the number of ‘G’s in a segment and then dividing the product by length of the segment. This method however falsely identifies the other G and C rich motifs, e.g., *Alu repeats*, as CGIs. In subsequent methods, these three conditions were made more stringent in order to reduce false identification at the expense of missing some true CGIs [24]. Sophisticated methods utilizing two Markov chain models [27,28], one for CGIs and the other for non-CGIs, are proposed [2,25,26]. These two Markov models differ in their respective model parameters which characterize the difference in transition probabilities between successive nucleotides in CGIs and non-CGIs, respectively. In these methods, a DNA segment is defined as CGI, if the log-score [2] computed using Markov model for a CGI is greater than that computed using Markov model for a non-CGI. Consequently, the model parameters used for CGIs and non-CGIs play a crucial role in identifying the CGIs. However, different methods employing such models from time-to-time produce inconsistent results. Another criterion based on the physical distance distribution of CpG dinucleoetides in a DNA segment has also been proposed [23]. Methods based on this criterion are dependent on nucleotide composition of a DNA sequence being analyzed and suffer from low identification specificity.

Recently, digital signal processing (DSP)-based algorithms have gained popularity for the analysis of genomic sequences since they can be mapped to numerical sequences. Digital filters have successfully been employed for identification of protein coding regions (exons) in DNA sequences and hot-spots in protein sequences [29-33]. Digital filters have also been used for identification of CGIs with considerable success [25,26]. These methods are similar to Markov chain methods but use digital filters to compute weighted log-score to identify CGIs. The method proposed in [25] employs a bank of IIR low-pass filters (about 40 filters, each with different bandwidth) to identify the CGIs by looking at the weighted log-scores of all the filters together. The CGI identification sensitivity of this method is affected by the tradeoff between responsiveness of filter and stability of the output. Moreover, this method may become computationally demanding as it makes use of a large number of filters in the bank. Another DSP based algorithm in [26] employs an underlying multinomial statistical model [34] to estimate its Markov chain parameters followed by an FIR filter with Blackman window to compute the weighted log-score.

It is evident from above discussion that the CGI identification methods and more importantly the criteria used therein play a crucial role in identifying CGIs. As such, development of fast and efficient computational methods with highly reliable CGI identification criteria is a necessity. Statistically optimal null filters (SONF) have been proven for their ability to efficiently estimate short-duration signals embedded in noise [35]. In this article, we propose a new DSP algorithm for identification of CGIs using SONF which combines maximum signal-to-noise ratio and least squares optimization criteria to estimate the message signal, characterizing the CGI, embedded in noise. Normally, the CGI identification accuracy is a lot dependent on the Markov models used and sometimes produces contrasting results. Also, one of the main objectives of the article is to find a uniform yet effective alternative CGI identification measure replacing the current measure based on transition probabilities. In the proposed scheme, we have formulated a simple basis function to be used in SONF which characterizes the CGI. Our criterion is devoid of any ambiguities associated with the choice of transition probabilities used in some of the algorithms. The proposed scheme is tested on a large number of already annotated DNA sequences obtained from human chromosomes 21 and 22. It is shown that our scheme is simple to implement and yet able to identify CGIs reliably and efficiently.

The rest of the article is organized as follows: the following section briefly describes a few existing DSP-based algorithms for the identification of CGIs. In Section “Proposed scheme”, the proposed SONF-based scheme for identifying CGIs in DNA sequences is explained. Results obtained from the proposed scheme are depicted as well as tabulated in Section “Results and discussion”. Finally, “Conclusion” section concludes the article describing some of the significant features of the proposed scheme.

## Related study

In this section, we give a brief review of some of the existing CGI identification methods as a preparatory groundwork for the method to be proposed in Section “Proposed scheme”.

### Markov chain approach

In this method, a DNA sequence of length *N*, represented as *X *= {*x*(*n*),*x*(*n* + 1),…,*x*(*n* + *N*−1)} where each symbol *x*(*n*)∈{*ACTG*} is considered as a first-order Markov chain [27] due to its conditional independence property, i.e., the nucleotide occurring at the location (*n*−1) does not offer any information over and above that at *n* to predict the nucleotide occurring at (*n* + 1). In a CpG island, the probability of transition from the nucleotide base C to the base G is higher in comparison with that in a non-CGI. Let the probability of transition from a nucleotide *β* to a nucleotide *γ* in a CGI and a non-CGI be denoted as $p_{\beta \gamma }^{+}$ and $p_{\beta \gamma }^{-}$ respectively. Tables 1 and 2 taken from [2] show the transition probabilities for CGI and non-CGI Markov models. These tables are derived from 48 putative CGIs in human DNA sequences. Each row in the tables contains transition probabilities from a specific nucleotide base to each of the four bases. These transition probabilities $p_{\beta \gamma }^{\pm }$ are calculated using

(1) $$p_{\beta \gamma }^{\pm }=\frac{n_{\beta \gamma }^{\pm }}{\underset{k\in \{A,T,G,C\}}{\sum }n_{\beta k}^{\pm }}$$

**Table 1:** Transition probabilities inside a CGI

$p_{\beta \gamma }^{+}$	**A**	**C**	**G**	**T**
A	0.180	0.274	0.426	0.120
C	0.171	0.368	0.274	0.188
G	0.161	0.339	0.375	0.125
T	0.079	0.355	0.384	0.182

**Table 2:** Transition probabilities inside a non-CGI

$p_{\beta \gamma }^{-}$	**A**	**C**	**G**	**T**
A	0.300	0.205	0.285	0.210
C	0.322	0.298	0.078	0.302
G	0.248	0.246	0.298	0.208
T	0.177	0.239	0.292	0.292

where $n_{\beta \gamma }^{\pm }$ is the number of dinucleoetides *βγ *in a DNA sequence. Naturally, every row in the tables adds up to unity. As expected, in Table 1, which corresponds to the CGI Markov model, the probability that a C is followed by a G is very high as compared with that in Table 2.

The CGIs in the DNA sequence *X* are identified by analyzing the windowed sequence *X*_*n *_= {*x*(*n*),*x*(*n* + 1),…,*x*(*n* + *L*−1)} of length *L*, and those obtained by shifting the window by one position at a time. The probability of observing a windowed sequence assuming that it belongs to a CGI is given by

(2) $$\begin{array}{l} P(X_{n}|\text{CGI})\;\\ =P(x(n)\ldots x(n+L-1)|x(n-1),\text{CGI}\;\text{model})\\ =\overset{L-1}{\underset{i=0}{\prod }}p_{x(n-1+i)x(n+i)}^{+} \end{array}$$

Similarly, the probability of observing this sequence assuming it belongs to a non-CpG island region is

(3) $$\begin{array}{l} P(X_{n}|\mathrm{non}\text{-}\mathrm{CGI})\\ =P(x(n)\ldots x(n+L-1)|x(n-1),\text{non}\text{-}\mathrm{CGI})\\ =\overset{L-1}{\underset{i=0}{\prod }}p_{x(n-1+i)x(n+i)}^{+} \end{array}$$

If the value of *P*(*X*_*n*_|CGI) >* P*(*X*_*n*_|non-*CGI*), then, it is concluded that the DNA sequence *X*_*n *_belongs to a CGI. Otherwise, it is more likely to be a non-CGI island. Alternatively, by formulating a log-likelihood ratio, given by

(4) $$S(n)=\frac{1}{L}\log\frac{P(X_{n}|\text{CGI})}{P(X_{n}|\text{non}\text{-}\mathrm{CGI})}$$

If *S*(*n*) > 0, the given DNA sequence is more likely to belong to a CGI, and if *S*(*n*) < 0 the sequence probably belongs to a non-CGI region.

### IIR low-pass filter approach

Yoon and Vaidyanathan [25] have noted that the log-likelihood ratio given in (4) can be expressed as:

(5) $$\begin{array}{l} S(n)=\frac{1}{L}\text{log}\overset{L-1}{\underset{n=0}{\prod }}\frac{p_{x(n-1)x(n)}^{+}}{p_{x(n-1)x(n)}^{-}}\\ \;=\frac{1}{L}\overset{L-1}{\underset{i=0}{\sum }}y(n+i)\\ \;=y(n)*h_{\text{ave}}(n) \end{array}$$

where *y*(*n*) is a sequence representing the log-likelihood ratio of a single transition given by

(6) $$y(n)=\text{log}\left(\frac{p_{x(n-1)x(n)}^{+}}{p_{x(n-1)x(n)}^{-}}\right)$$

and, *h*_*ave*_(*n*) is a simple averaging filter defined as

(7) $$h_{\text{ave}}(n)=\left\{\begin{array}{ll} 1/L, & \text{for}-L+1\leq n\leq 0\\ 0, & \text{otherwise}. \end{array}\right)$$

Then, they proposed using a bank of *M* filters each having different bandwidth, instead of using simply one low-pass filter *h*_ave_(*n*). Specifically, the filter used in the *k*th (*k *= 0,…,*M*−1) channel has a transfer function given by

(8) $$H_{k}(z)=\frac{1-\alpha_{k}}{1-\alpha_{k}z^{-1}}$$

where 0 <* α*_0_ <* α*_1_ < ⋯ <* α*_*M*−1_ < 1. Since impulse response of a filter in the bank is $h_{\text{ave}}\left(k\right)=\left(1,-,\alpha_{k}\right)\alpha_{k}^{k}u\left(n\right)$ more recent inputs are given larger weights than the past ones in the averaging process of *y*(*n*). The filter bank consists of 40 channels (*M *= 40), and the filter parameter *α*_*k *_is chosen from 0.95 to 0.99 with an increment of 0.001. The log-likelihood ratio obtained from the output of the *k*th channel is given by

(9) $$S_{k}(n)=y(n)*h_{k}(n)$$

The values of *S*_*k*_(*n*) obtained for all *k* and *n* are then used to obtain a two-level contour plot. The bands corresponding to *S*_*k*_(*n*) > 0 determine the locations of CGIs.

In this method, the use of filter bank increases the computational overhead considerably. For fair comparison, instead of a bank on *M* filters, we have used one pole filter with optimized parameter *α *= 0.99 to compare with other methods (this reduces the number of computations considerably).

### Multinomial statistical model

This method by Rushdi and Tuqan [26] differs from the previous method by the way the transition tables are obtained and the type of digital filter used to calculate the log-likelihood ratio. Instead of using (1) to obtain the transition probability tables, they are generated by comparing the frequency of each dinucleotide with the one expected under a multinomial model [34]. Transition probabilities $p_{\beta \gamma }^{\pm }$ for the windowed sequence *X*_*n *_are calculated using

(10) $$p_{\beta \gamma }^{\pm }=\frac{c_{\beta \gamma }^{\pm }}{\underset{k\in \{A,T,G,C\}}{\sum }c_{\beta k}^{\pm }}$$

where

(11) $$c_{\beta \gamma }^{\pm }=\frac{{\text{frequency}}_{\beta \gamma }^{\pm }(n)}{\left({\text{frequency}}_{\beta }^{\pm }(n))({\text{frequency}}_{\gamma }^{\pm }(n)\right)}$$

This method uses a FIR digital filter with variable coefficients generated by Blackman window to calculate the log-likelihood ratio *S*(*n*) given in (4). The locations with *S(n)* greater than zero are the probable locations of CGIs.

All of the above-mentioned methods rely on the transition probability tables to calculate log-likelihood ratio used to identify CGIs. The methods [25,26] specifically vary by the way *y*(*n*), obtained from the respective transition tables, are averaged. It is shown later in Section “Results and discussion” that the choice of the transition tables may produces contrasting results. Hence, a more reliable and efficient scheme that is devoid of these transition tables is necessary for identifying CGIs.

## Proposed scheme

**Figure 2:**
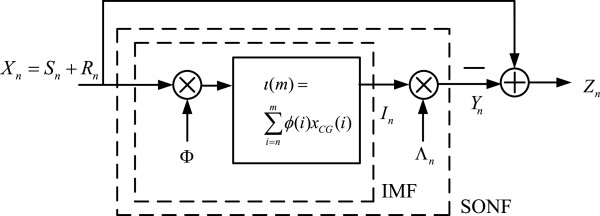
Statistically optimal null filter.

In this study, we adopt the SONF approach, proposed in [35], to efficiently identify CGIs in DNA sequences. SONF is used for estimation of short duration signal, *S*_*n *_= {*s*(*m*)}, embedded in noise *R*_*n *_= {*r*(*m*)} by combining maximum signal-to-noise ratio and least squares optimization criteria. The implementation of the twofold optimization in SONF is shown in Figure 2, where an instantaneous matched filter (IMF) is first used to detect the presence of a short duration signal embedded in noise by maximizing the signal-to-noise ratio over variable-time observation interval *m*. The IMF output, *I*_*n*_, is then scaled by a locally generated function *⋀*_*n*_, by least squares (LS) optimization procedure, to obtain the signal *Y*_*n*_, an estimate of *S*_*n*_. It has been shown that the SONF is equivalent to a Kalman filter with a much simpler implementation [35]. Also, SONF has the ability to track rapidly changing signals leading to more practical processing of short-duration signals [36,37]. Therefore, the proposed scheme is expected to perform better in situations even if the CGIs are of very short length of the order of 200 bp.

To be able to apply SONF approach to identify CGIs, the DNA sequence *X*, of length *N*, is first mapped to an appropriate binary numerical sequence *X*_*CG *_= {*x*_*CG*_(*n*)}. A sliding window of length *L* is used to evaluate if each of the windowed sequences, *X*_*n *_= {*x*_*CG*_(*m*)}, where *n *= 1,2,…,*N*−*L* + 1 and *m *=* n*,*n* + 1,…,*n* + *L*−1, belong to a CGI or not. Each of the windowed sequence *X*_*n *_can be expressed as

(12) $$X_{n}=S_{n}+R_{n}$$

where *S*_*n *_= {*s*(*m*)} is a message signal corresponding to a CGI and *R*_*n *_= {*r*(*m*)} is a residual signal. *S*_*n *_and *R*_*n*_ are each of length *L*. Let Φ = {*ϕ*(*m*)} be a fixed binary basis sequence of length *L* having some characteristic property of CGI.

Now, the message signal corresponding to a CGI can be expressed as *S*_*n *_=* V*_*n*_Φ, where *V*_*n *_= {*v*(*m*)} and Φ are sequences each of length *L*. The sequence *V*_*n*_Φ is obtained by multiplying the corresponding elements of *V*_*n *_and Φ. The sequence *V*_*n*_ is determined by minimizing *R*_*n*_ in least square sense. Let the message signal be denoted as *S*_*n *_= {*s*(*m*)}. The objective of the proposed method is to choose the basis sequence such that *V*_*n*_ resulting from the optimization process has some discriminating feature of indicating whether the associated sequence *X*_*n*_ belongs to a CGI. The following subsections explain in detail the steps involved in identification of CGIs in a DNA sequence using SONF.

### Numerical mapping of DNA sequences

As DNA sequences are alphabetical in nature, they need to be mapped to numerical sequences in order to employ the DSP techniques for DNA sequence analysis. There are several mapping techniques reported in the literature. One of the earliest and a popular mapping is that of Voss’s binary indicator sequences [38]. A DNA sequence *X* can be mapped to a set of four digital signals by forming four binary indicator sequences, namely, *X*_*A*_, *X*_*T*_, *X*_*G*_, and *X*_*C*_. In each of these binary indicator sequences, ’1’ represents the presence and ’0’ absence of the corresponding bases A, T, G, and C in *X*. For instance, considering a DNA sequence *X *= {*ATCCGAAGTATAACGAA*}, the binary indicator sequence corresponding to G, i.e., *X*_*G *_can be expressed as *X*_*G *_= {00001001000000100}. Indicator sequences for the remaining three nucleotides can be represented in a similar fashion.

The problem of CGI identification deals with G and C content in a DNA sequence. Hence, we define a new indicator sequence *X*_*CG *_= {*x*_*CG*_(*n*)}, which indicates the presence of the nucleotides C and G in the DNA sequence. For example, the binary indicator sequence *X*_*CG*_ of the DNA sequence above is *X*_*CG *_= {00111001000001100}.

### Choosing the basis sequence

**Figure 3:**
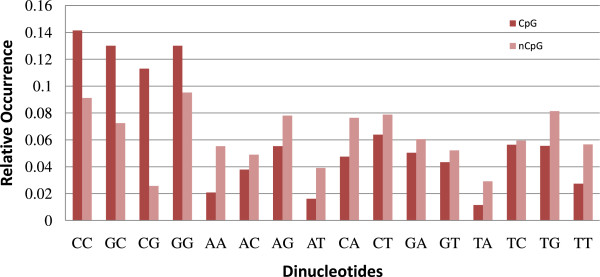
Comparison of relative occurrence of dinucleotides in CGIs and non-CGIs of L44140.

In this study, we have noticed that the dinucleotides CC, CG, GC, and GG occur more frequently in a CGI as compared to a non-CGI. For this study, we have calculated the occurrence of these four dinucleotides in the sequence L44140 taken from the chromosome X of *Homo sapiens*. The sequence L44140 is of length 219447 bp and has 17 CGIs whose locations are obtained from [39]. Figure 3 depicts the relative occurrence of the above four dinucleotides as compared to the remaining dinucleotides (AA, AC, AG, AT, CA, CT, GA, GT, TC, TG, TT, and TA) in CGIs and non-CGIs of L44140. Here, the relative occurrence of a particular dinucleotide is equal to the number of times that dinucleotide occurs in the sequence divided by the sequence length. It is evident that the dinucleotides CC, CG, GC, and GG occur more frequently in CGIs whereas the other dinucleotides occur more frequently in non-CGIs. This observation can also be inferred from the transition probability tables (Tables 1 and 2) as the values of $p_{\beta \gamma }^{+}$ are greater than $p_{\beta \gamma }^{-}$, where *β *and *γ* are either G or C. In Figure 3, the darker bars corresponding to the dinucleotides CC, CG, GC, and GG are taller in CGIs, whereas the darker bars corresponding to the other dinucleotides are shorter. Hence, instead of just considering the difference in relative occurrence of CG, it is more productive to consider the relative occurrence of the dinucleotides CC, CG, GC, and GG to distinguish between a CGI and a non-CGI.

**Figure 4:**
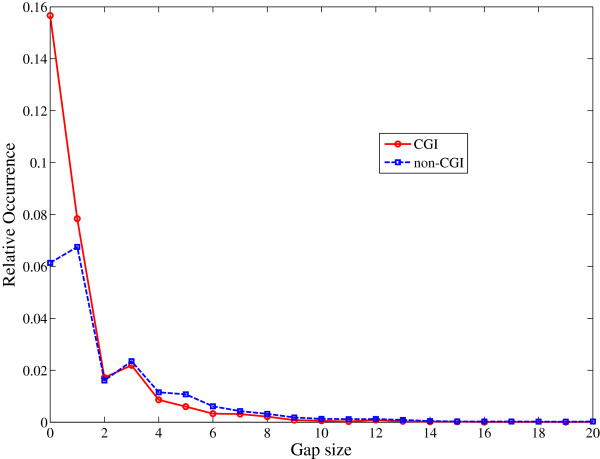
Relative occurrence of various gap sizes in CGIs and non-CGIs of L44140.

Moreover, we have studied the difference in gap sizes between the dinucleotides CC, CG, GC, and GG in CGIs and non-CGIs of L44140. The shortest possible gap is of size 0 when the dinucleotides are adjacent to each other. Figure 4 shows the relative occurrence of gaps of various sizes in a CGI and a non-CGI. Here, relative occurrence of a particular gap size is equal to the number of times that gap size occurs in the sequence divided by the sequence length. Obviously, the gap of size 0 occurs more frequently in a CGI as compared to that of a non-CGI. And, it is found that the gap size in a non-CGI can go up to 40 where as in CGIs the maximum gap size was found to be 19. It can also be seen that the gaps of sizes 0, 1, and 2 occur more frequently in a CGI and the gap sizes of 3 and greater occur more frequently in a non-CGI. A gap of size 2 is the largest gap which can distinguish between a CGI and a non-CGI.

Based on the above observations, the basis sequence which characterizes a CGI can be formulated as Φ = {1100110011…001100}. The 1’s in Φ represent either the nucleotide C or G. The 1’s always appear in pairs where each pair representing one of the dinucleotide CC, CG, GC, or GG. The 0’s in Φ form the gap between the dinucleotides. A gap size of 2 is chosen between the dinucleotides. This choice of Φ is also satisfies the basic criteria of a CGI, i.e., at least 50% of the nucleotide content in a CGI is due to C and G.

**Figure 5:**
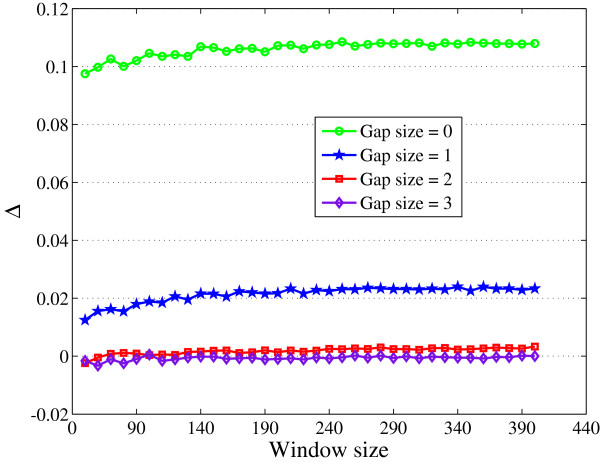
Difference of relative occurrence of a particular gap in a CGI and a non-CGI for different window lengths.

Now, in order to obtain the length of Φ (window size), we have analyzed CGIs and non-CGIs of different lengths for the relative occurrence of various gap sizes. Figure 5 shows the plot of Δ* versus* window size for various gap sizes. Here, Δ is the difference of relative occurrence of a particular gap in a CGI and a non-CGI for a fixed window length. It can be seen that Δ is maximum for gap size 0. As the window size increases Δ also increases before it reaches a steady value. Δ is negative for gap sizes of 3 and greater signifying that the gap sizes of 3 and higher are more probable in non-CGIs compared to CGIs. For the gap size 2, Δ stabilizes for window sizes greater than 200. Larger the window size, larger the number of computations, and hence in the proposed method we have used the length of Φ (window size) to be equal to 200.

### IMF

The objective of IMF, which is the first stage of SONF shown in Figure 2, is to detect the presence of the waveform Φ in the input sequence *X*_*n*_. IMF is an improvement over a matched filter, the difference being, in IMF optimal SNR is repeatedly calculated at every sample *m*, over an observation interval *m*∈[*n*,*n* + *L*−1]. IMF takes *X*_*n*_ and Φ as inputs and produces an output sequence *I*_*n *_= {*ι*(*m*)} where

(13) $$\iota (m)=\overset{m}{\underset{i=n}{\sum }}x_{\text{CG}}(i)\phi (i)$$

for *m *=* n*,*n* + 1,…,*n* + *L*−1. It can be seen that at each sample *m*, *ι*(*m*) is calculated over a varying interval *i*∈[*n*,*m*]. Note that, assuming *ι*(*n*−1) = 0, *ι*(*m*) can also be calculated using the recursive relation given by

(14) $$\iota (m)=\iota (m-1)+x_{\text{CG}}(m)\phi (m).$$

The output *ι*(*m*) leads to an optimal detection of Φ at each sample *m*, and can be expressed as

(15) $$\iota (m)=v(m)c(m)+r_{0}^{\prime }(m)$$

where $r_{0}^{\prime }(m)$ is the residual signal in IMF output, and *c*(*m*) is given by

(16) $$c(m)=\overset{m}{\underset{i=n}{\sum }}\phi^{2}(i).$$

The *v*(*m*)∈*V*_*n *_in (15) is an unknown gain.

### Least square optimization of the IMF output

The objective of the second stage in SONF is to determine a sequence *⋀ *= {*λ*(*m*)}, which when used to scale the IMF output *I*_*n*_, produces the SONF output, *Y*_*n*_, such that $Y_{n}\to V_{n}\Phi$. Here, *Y*_*n*_ is an element wise product of *V*_*n *_and Φ. *Y*_*n*_ is an estimate of *S*_*n*_, which is the message signal corresponding to CGI.

Let us consider the suboptimal case in which a sample of the IMF output *ι*(*m*) in (15), when scaled by *λ*(*m*)=*ϕ*(*m*)/*c*(*m*), generates

(17) $$\begin{array}{l} y(m)=v(m)c(m)+r_{0}^{\prime }\frac{\phi (m)}{c(m)}\\ \;\;=v(m)\phi (m)+r_{0}(m)\\ \;\;=s(m)+r_{0}(m) \end{array}$$

where *y*(*m*) is an element of the SONF output, *Y*_*n*_. As we desire optimal null filtering, i.e., *y*(*m*) =* s*(*m*), the residual element, *r*_0_(*m*), needs to be entirely eliminated.

Before determining the optimal *⋀*_*n*_, corresponding to ideal null filtering, we define the sequence *Z*_*n *_= {*z*(*m*)} such that,

(18) $$\begin{array}{l} z(m)=x_{\text{CG}}(m)-y(m)\\ \;\;=s(m)+r(m)-\lambda (m)\iota (m) \end{array}$$

Ideally, *y*(*m*) =* s*(*m*) and from (18), *z*_ideal_(*m*) =* r*(*m*). Now, the optimal *⋀*_*n*_={*λ*_opt_(*m*)} is determined by minimizing the mean square error, $E[e_{\lambda }^{2}(m)]$, with respect to *λ*(*m*) where

(19) $$e_{\lambda }(m)=z_{\text{ideal}}(m)-z(m).$$

The optimal post IMF scaling sequence *λ*_opt_(*m*) obtained by carrying out the above mean square minimization is [35]

(20) $$\lambda_{\text{opt}}(m)=\frac{\phi (m)}{c(m)+1/\text{SNR}}$$

where SNR is the input signal-to-noise ratio (considering *r*(*m*) to be noise).

In order to implement SONF, the value of the input SNR should be known. To circumvent this problem, a suboptimal case, as shown in (17), is assumed considering *c*(*m*) >> 1/SNR, leading to

(21) $$\lambda_{\text{subopt}}(m)\overset{}{\to }\frac{\phi (m)}{c(m)}$$

It can be shown that as *m* increases, $\lambda_{\text{subopt}}(m)\to \lambda_{\text{opt}}(m)$ because the second term in the equation

(22) $$\frac{\lambda_{\text{subopt}}(m)}{\lambda_{\text{opt}}(m)}=1+\frac{1}{\left(\text{SNR})c(m\right)}$$

approaches zero (as the value of *c*(*m*) progressively increases with *m*). So, the value of initial SNR in (20) will influence only the starting few samples in *Y*_*n*_.

The SONF can easily be implemented recursively using the following equations [35]

(23) $$\begin{array}{l} \iota (m)=\iota (m-1)+x_{\text{CG}}(m)\phi (m)\\ P(m)=P(m-1)-\frac{P(m-1)\phi (m)\phi (m)P(m-1)}{1+\phi (m)P(m-1)\phi (m)}\\ \lambda (m)=P(m)\phi (m)\\ y(m)=\iota (m)\lambda (m) \end{array}$$

In this case of DNA analysis, one may choose the initial value of the gain *P*(0) to be 1 and *ι*(0) =* ι*(1).

The proposed SONF-based CGI identification algorithm for a DNA sequence of length *N* can now be summarized as follows:

**Initialization:** Set the base location index *n *= 0. 

•** Step 1:** Apply a rectangular window of length *L *= 200 starting at the base location *n* of the DNA sequence *X* to obtain the windowed sequence *X*_*n*_.

•** Step 2:** Obtain the binary indicator sequence *X*_*CG *_for the windowed sequence, *X*_*n*_, from Step 1.

•** Step 3:***X*_*CG *_from Step 2, along with the binary basis sequence Φ, form the inputs to SONF. The corresponding SONF output sequence, *Y*_*n*_, is evaluated using the recursive relations given in (23), by assuming *P*(0) = 1 and *ι*(0) =* ι*(1).

•** Step 4:** Compute the SNR power gain *G*(*X*_*n*_), which is the ratio of the variance of the SONF output, *Y*_*n*_, to the variance of the corresponding input *X*_*n*_.

•** Step 5:** Increment the value of *n* by 1, i.e., *n *=* n* + 1. If *n *≤ (*N*−*L*) go to Step 1, else go to Step 7.

•** Step 6:** Plot *G*(*X*_*n*_) as a function of *n* + *L *and get its upper envelope. The peaks in the resulting plot which are above the threshold, *η*, indicate the locations of CGIs identified in *X*.

•** Step 7:** Exit the algorithm.

**Figure 6:**
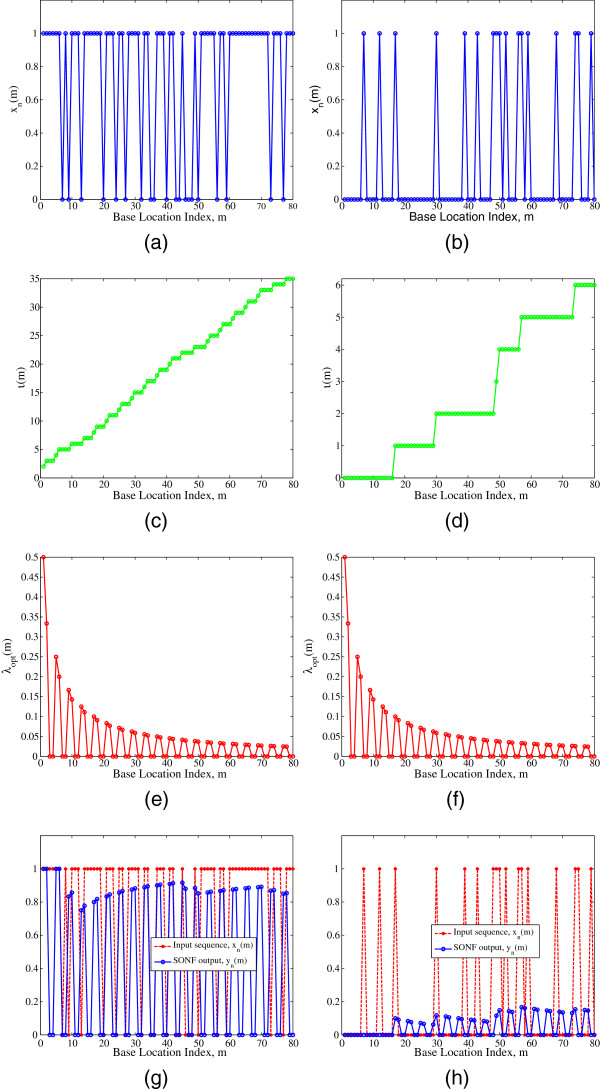
SONF implementation: (a) An example of a CGI; (b) An example of a non-CGI; (c) IMF output for CGI; (d) IMF output for non-CGI; (e) Scaling function for CGI; (f) Scaling function for non-CGI; (g) SONF output for CGI; and, (h) SONF output for non-CGI.

Figure 6 shows the SONF implementation for better understanding of the proposed approach. Figure 6a,b shows an example of a CGI and a non-CGI with 80 bp. Naturally, in Figure 6a there are greater number of ones. Figure 6c,d shows the IMF output for a CGI and a non-CGI, respectively. It can be seen that the IMF output corresponding to a CGI progressively increases to a greater value of 35 as compared to 6 of that of a non-CGI. Figure 6e,f is the scaling functions for a CGI and a non-CGI, respectively. They are obtained using the relation *λ*(*m*) =* P*(*m*)*ϕ*(*m*) in (23). Finally, Figure 6g,h shows the estimated CGI characteristic in a CGI and a non-CGI, respectively. The SONF output corresponding to a CGI has greater amplitude as compared with that of a non-CGI.

### Prediction measures

**Figure 7:**
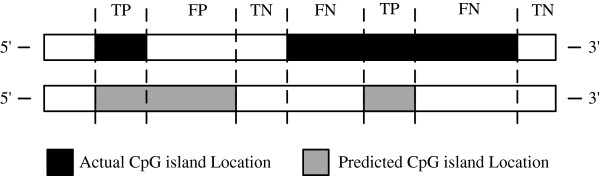
Four possible outcomes of CGI prediction.

The identification of CGIs can have four possible outcomes; true positive (TP), true negative (TN), false positive (FP), or false negative (FN) as shown in Figure 7. Two basic measures of determining the accuracy of prediction are sensitivity (Sn) and specificity (Sp) [40]. Sensitivity, given by

(24) $$\text{Sn}=\frac{\text{TP}}{\text{TP}+\text{FN}}$$

and is defined as the proportion of CGIs that have been predicted correctly. Whereas, specificity given by

(25) $$\mathrm{Sp}=\frac{\text{TP}}{\text{TP}+\mathrm{FP}}$$

is defined as the proportion of the predicted CGIs that are real. Sensitivity and specificity can take on values from 0 to 1. For a perfect prediction, Sn = 1 and Sp = 1. Neither sensitivity nor specificity alone can provide a good measure of the global accuracy, because high sensitivity can be achieved with little specificity and vice versa. A measure that combines sensitivity and specificity values is called the correlation coefficient (CC) and is given by

(26) $$\text{CC}=\frac{\left(\text{TP}\times \text{TN})-(\text{FN}\times \text{FP}\right)}{\sqrt{\left(\text{TP}+\text{FN})(\text{TN}+\text{FP})(\text{TP}+\text{FP})(\text{TN}+\text{FN}\right)}}$$

The value of CC ranges from −1 to 1, where a value of 1 corresponds to a perfect prediction; a value of −1 indicates that every CGI has been predicted as non-CGI, and vice versa.

Another measure, called the performance accuracy (Acc), used in our analysis is given by

(27) $$\text{Acc}=\frac{\text{TP}+\text{TN}}{\text{TP}+\text{FP}+\text{TN}+\text{FN}}$$

In this article, we have evaluated the performance of different CGI identification methods at the nucleotide level. For example, the value of TP is obtained by adding all the nucleotides predicted to to true positive, and the other outcomes are calculated in the similar manner. At the CGI level, even if one nucleotide (or a threshold of a minimum number of nucleotides) corresponding to a CGI is predicted to be true positive the entire CGI is assumed to be predicted correctly.

## Results and discussion

The proposed CGI prediction scheme is tested on several genomic sequences of varying lengths taken from the human chromosomes 21 and 22. More precisely, we have used the three contigs, NT_113952.1, NT_113954.1, and NT_113958.2 from chromosome 21, and the contig NT_028395.3 from chromosome 22 for our analysis. All the sequence data considered for this study are obtained from the GenBank Database [39]. The performance of the proposed scheme is compared with the other popular DSP-based approaches such as Markov chain [2], IIR low-pass filters [25], and multinomial model [26].

First, a DNA sequence from human chromosome X with the GenBank accession number of L44140 is analyzed for illustrative purpose. The sequence is of length 219447 bp and is already annotated, i.e., the locations of its CGIs are already known and can be obtained from [39]. The sequence L44140 is also used to obtain the values of threshold, *η*, used by the DSP-based methods being compared in this article.

**Figure 8:**
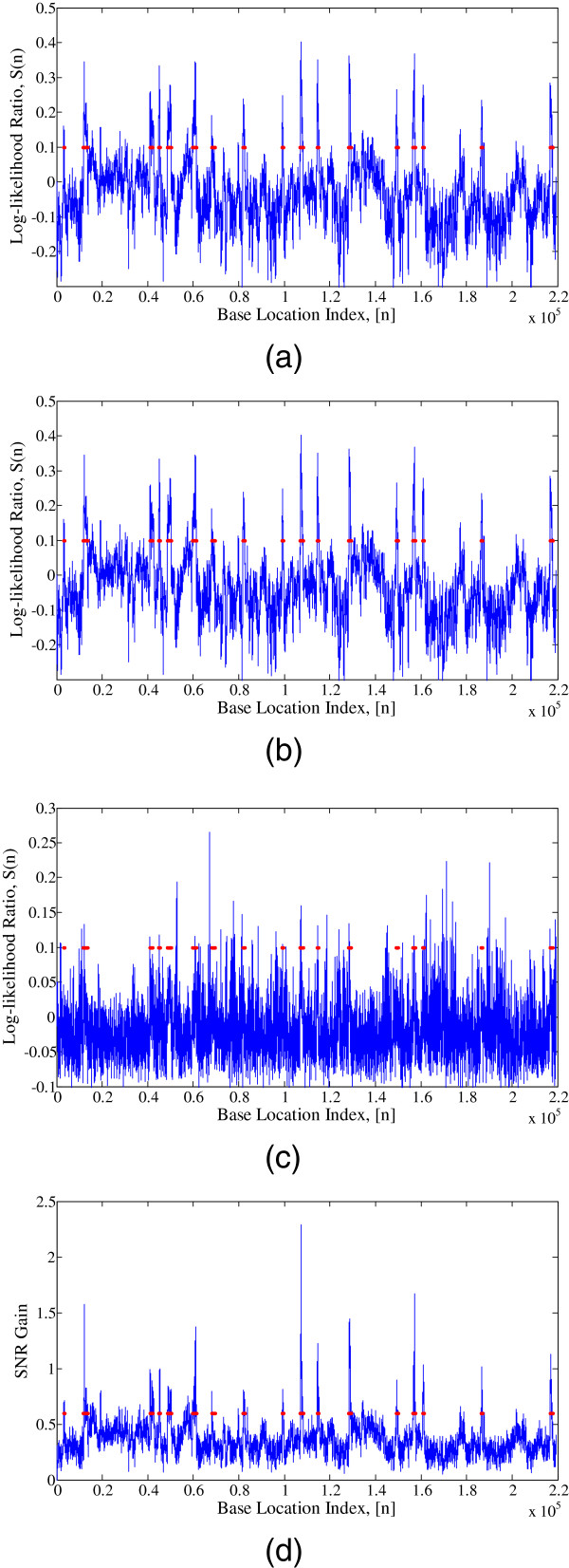
CGI prediction in the DNA sequence L44140 using (a) Markov chain method; (b) IIR Filter method; (c) Multinomial model; (d) SONF scheme.

Figure 8 shows the comparative performance of CGI prediction by the above-mentioned four approaches. Figure 8a shows the performance of Markov chain approach, where log-likelihood ratio *S*(*n*) is plotted against base index of the sequence. The transition probability tables given in Tables 1 and 2 are used to calculate *S*(*n*). All the base locations, *n*, with *S*(*n*) > 0 imply that they are very likely to be a part of a CGI. A window length of 200 bp is considered for the method. Markov chain method is able to detect most of the CGIs in the DNA sequence and it can be seen that the CGIs and non-CGIs can reasonably be differentiated by looking at the sign of *S*(*n*). However, one of the major drawbacks of this method is the presence of a lot of false positives that falsely categorize non-CGIs into CGIs.

Figure 8b shows the performance of IIR low-pass filter approach where the log-likelihood ratio, *S*(*n*), is plotted against base index of the sequence, *n*. The transition probability tables given in [25] are used to calculate *S*(*n*). For fair comparison, instead of a bank on *M* filters, we have used one pole filter with optimized parameter *α *= 0.99 for this method. All the base locations, *n*, with *S*(*n*) > 0 imply that they are very likely to be a part of a CGI. A window length of 200 bp is considered for the method. Similar to the Markov chain method, this method also produces a lot of false positives affecting the prediction accuracy.

Figure 8c shows the prediction of CGIs using the multinomial model in [26]. An underlying multinomial statistical model is employed to estimate the Markov chain model parameters that result in the transition probability tables given in [26]. A Blackman window of length 100 bp is employed for calculating the filtered log-likelihood ratio. The Blackman window gives larger weights for central samples of the window, thus reducing the edge effects. Windows with the positive filtered log-likelihood ratio are considered to be a part of a CGI. This method shows considerably high false positives making the CGI prediction unreliable.

Figure 8d shows performance of the proposed SONF scheme in predicting the CGIs. Unlike the above-mentioned methods, our scheme utilizes the binary basis sequence, Φ, instead of the probability transition tables. The proposed scheme first maximizes SNR of the output at each time instant using IMF, then it further enhances the estimated signal using least-square optimization criterion, to estimate the presence of Φ in the input windowed DNA sequence. A window size of 200 is used for the proposed method. Effectiveness of the proposed scheme is clearly visible in Figure 8d, which depict more contrasting peaks as compared to the other three approaches. These contrasting peaks make the identification process comparatively easier resulting in less number of false positives.

**Figure 9:**
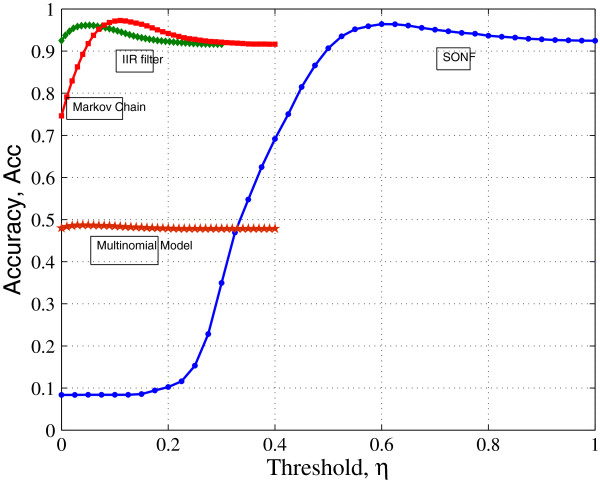
Relation between the performance Acc and threshold.

**Figure 10:**
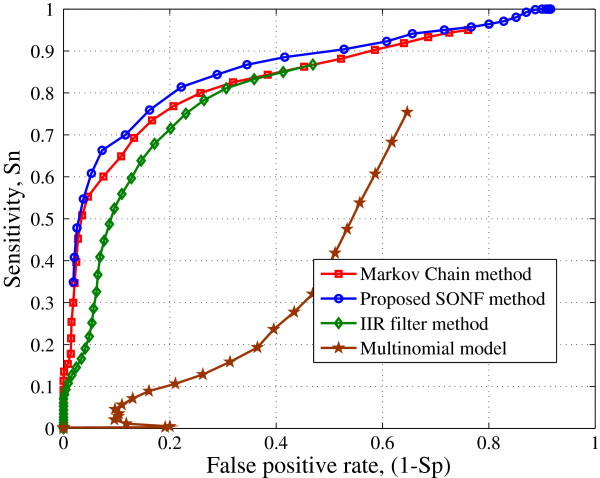
ROC curves obtained for the sequence L44140.

It can be seen from Figure 8 that the default threshold on *η *= 0 produces a lot of false positives for the methods using transition probability tables. The optimal threshold values for the methods is obtained by calculating the prediction Acc for varying thresholds for each method (Figure 9). The optimal values of thresholds obtained for the Markov chain method, IIR filter method, and the proposed SONF approach are 0.1, 0.05, and 0.6, respectively. The actual locations of the CGIs, obtained from NCBI website, present in the sequence L44140 are represented by red horizontal spots in Figure 8. Figure 10 is receiver operating characteristic (ROC) curves plotted for the four methods. It can be seen that the proposed approach has better overall performance for the sequence L44140 with the area under the curve 0.7460. The Markov chain method is next with the area under the ROC curve 0.6072. The area under the curve for IIR filter method is 0.3106. It can be seen that the multinomial model method has the least area under the ROC curve. The dismal performance of the multinomial model does not indicate anything about the method in itself but merely implies that the transition probability tables used may not be appropriate for the example considered.

We have evaluated the time complexity of the proposed method using the *tic-toc* function in MATLAB. Taking the necessary precautions (such as all applications except MATLAB were closed, a fresh session of MATLAB was started for each task, and MATLAB was warmed up with the code, i.e., the first run of the code was ignored), the CPU time for processing a fixed length of sequence, the Markov chain method was found to be the least followed by SONF, IIR and multinomial approaches with an additional CPU time of 1.29%, 1.78%, and 1.82%, respectively. This difference is not substantial considering today’s computing resources.

**Figure 11:**
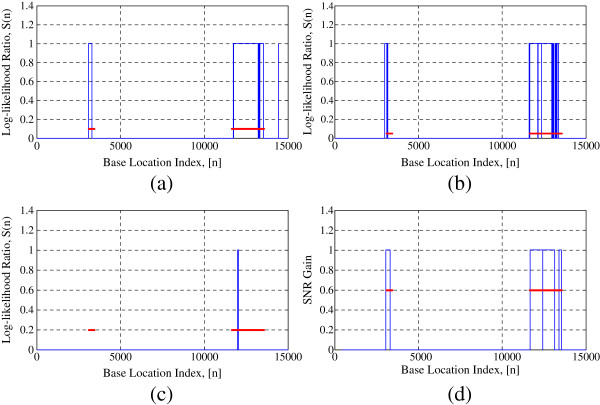
**CGI prediction in the first 15000 bps of L44140 using (a) Markov chain method; (b) IIR filter method; (c) multinomial model; (d) SONF scheme.** Binary decision based on respective threshold is plotted against the base location index

Figure 11 shows the performance of the four methods for the prediction of CGIs in the first 15000 bps of L44140. The red horizontal lines are the actual locations of CGIs. The blue binary decision curve depicts the locations of the predicted CGI by the methods. As can be seen from Figure 11c, the multinomial-based approach fails to detect the CGI located between base pairs 3095 and 3426 as opposed to other three methods implying that the probability transition parameters used for the CGI identification play a crucial role. Hence, it is important to have a CGI identification characteristic which is devoid of any ambiguity with the choice of different probability transition tables available. The binary basis sequence Φ in the proposed scheme successfully identifies the CGIs and can be reliably used as CPG identification characteristic.

Table 3 presents the summary of performance measures Sn, Sp, CC, and Acc obtained for the analysis of four contigs NT_113952.1, NT_113954.1, NT_113958.2, and NT_028395.3. The performance of the proposed scheme is also compared with that of CpGCluster [23], which uses the distance between CpG dinucleotides (and not the transition probability tables) for identifying CGIs. The proposed approach has the highest values of Sn for all the contigs (shown in bold) and has the highest values of CC for the contigs NT_113954.1 and NT_113958.2. The performance accuracy is also quiet high, consistently above 97% which is a good sign. This shows that the proposed method is reliable and the proposed binary basis sequence Φ is an alternative CGI identification characteristic. The multinomial method did not identify any of the CGIs in the contig NT_028395.3 and hence its Sn and Sp values are zero. The corresponding Acc value is high because the method predicting most of the true negatives correctly. The contig NT_028395.3 has short CGIs of the order of 200 bps and the proposed approach with better sensitivity is capable of identifying them.

**Table 3:** Comparison of different methods for identification of CGIs

**Contig.**	**Performace**	**Methods**				
		**Markov Chain**	**IIR Filter**	**Multinomial model**	**CpGCluster**	**SONF**
**NT_113952.1**	Sn	0.8466	0.8656	0.4524	0.5046	**0.8677**
*Length = 184355*	Sp	0.8728	0.8320	0.2833	0.9995	0.4457
	CC	0.8621	0.8180	0.3609	0.6941	0.6192
	Acc	0.9955	0.9848	0.4948	0.9778	0.9878
**NT_113954.1**	Sn	0.3285	0.2226	0.0055	0.2986	**0.5420**
*Length = 129889*	Sp	0.3082	0.2585	0.0021	0.9946	0.2094
	CC	0.3152	0.2369	0.0040	0.4381	0.4382
	Acc	0.9940	0.9940	0.4989	0.9690	0.9894
**NT_113958.2**	Sn	0.4555	0.3561	0.2938	0.2716	**0.8852**
*Length = 209483*	Sp	0.4652	0.4439	0.0202	0.9994	0.2880
	CC	0.4527	0.3899	0.0119	0.4996	0.4954
	Acc	0.9849	0.9845	0.4960	0.9532	0.9705
**NT_028395.3**	Sn	0.5440	0.4200	0.0000	0.4489	**0.8789**
*Length = 647850*	Sp	0.8233	0.7590	0.0000	0.9947	0.4534
	CC	0.6667	0.5616	-0.0116	0.9753	0.6267
	Acc	0.9945	0.9932	0.8710	0.9532	0.9887

## Conclusion

In this article, a new DSP-based technique using SONFs is proposed for the prediction of CGIs in DNA sequences. A novel CPG identification characteristic is presented in the form of a binary basis sequence which is shown to identify CGIs reliably. It has also been shown that the performance of the existing methods which use discriminating transition probability tables for CGIs/non-CGIs is not consistent. The prediction accuracy of these methods are highly dependent on the training data used to obtain the transition probabilities of CGIs and non-CGIs. The inability of finding a unique CGI identification characteristic has resulted in failure in predicting many of the CGIs. This article makes an attempt to present a unique CGI identification characteristic which does not require any training. Furthermore, the ability of SONF to track short duration signals is exploited in identifying the CGIs in DNA sequences. SONF combines maximum signal-to-noise ratio and least squares optimization criteria to estimate the CGI identification characteristic in the DNA sequence. The performance of the proposed technique is tested on four randomly chosen contigs in chromosomes 21 and 22 of human beings. The simulation results comparing the performance of the proposed technique with the other three DSP-based CGI prediction techniques have shown that the proposed approach enjoys superior prediction accuracy in terms of sensitivity. The overall predicting accuracy of the proposed approach is also consistently above 97% and is comparable to that of the Markov chain method making it a reliable method.

## Competing interests

The authors declare that they have no competing interests.
